# Recommendation of a practical guideline for safe tracheostomy during the COVID-19 pandemic

**DOI:** 10.1007/s00405-020-05993-x

**Published:** 2020-04-21

**Authors:** Arunjit Takhar, Abigail Walker, Stephen Tricklebank, Duncan Wyncoll, Nicholas Hart, Tony Jacob, Asit Arora, Christopher Skilbeck, Ricard Simo, Pavol Surda

**Affiliations:** 1 Department of Otorhinolaryngology-Head and Neck Surgery, Guy’s and St Thomas’ NHS Foundation Trust, Great Maze Pond, London, SE1 9RT UK; 2 Department of Otorhinolaryngology-Head and Neck Surgery, University Hospital Lewisham NHS Trust, Lewisham High Street, London, SE13 6LH UK; 3 Department of Critical Care, Guy’s and St Thomas’ NHS Foundation Trust, Westminster Bridge Road, London, SE1 7EH UK; 4 Lane Fox Respiratory Service, Guy’s and St Thomas’ NHS Foundation Trust, Westminster Bridge Road, London, SE1 7EH UK

**Keywords:** SARS-CoV-2, Novel coronavirus, COVID-19, Tracheostomy, Mechanical ventilation

## Abstract

**Purpose:**

The COVID-19 pandemic is placing unprecedented demand upon critical care services for invasive mechanical ventilation. There is current uncertainty regarding the role of tracheostomy for weaning ventilated patients with COVID-19 pneumonia. This is due to a number of factors including prognosis, optimal healthcare resource utilisation, and safety of healthcare workers when performing such a high-risk aerosol-generating procedure.

**Methods:**

Literature review and proposed practical guideline based on the experience of a tertiary healthcare institution with 195 critical care admissions for COVID-19 up until 4th April 2020.

**Results:**

A synthesis of the current international literature and reported experience is presented with respect to prognosis, viral load and staff safety, thus leading to a pragmatic recommendation that tracheostomy is not performed until at least 14 days after endotracheal intubation in COVID-19 patients. Practical steps to minimise aerosol generation in percutaneous tracheostomy are outlined and we describe the process and framework for setting up a dedicated tracheostomy team.

**Conclusion:**

In selected COVID-19 patients, there is a role for tracheostomy to aid in weaning and optimise healthcare resource utilisation. Both percutaneous and open techniques can be performed safely with careful modifications to technique and appropriate enhanced personal protective equipment. ORL-HNS surgeons can play a valuable role in forming tracheostomy teams to support critical care teams during this global pandemic.

## Introduction

The COVID-19 pandemic is placing unprecedented demands upon critical care services across the world. As of the 10th April 2020, there are currently 70,272 confirmed cases in the UK and over 1.6 million globally, with around half these total cases in Europe [1]. Current evidence from China suggests that between 9.8 and 15.2% of patients will require invasive mechanical ventilation or extracorporeal membrane oxygenation (ECMO) [2–4]. On 10th April 2020, the intensive care national audit and research centre (ICNARC) published their second report on data from 3883 confirmed COVID-19 admissions to critical care in England, Wales and Northern Ireland. Of these, 1689 were completed episodes with 871 deaths and 818 discharged from critical care, giving a present mortality rate of 51.6% for those requiring critical care admission [5]. As a high consequence infectious disease unit, our institution was one of the first in the UK to treat confirmed COVID-19. As of 4th April 2020, we have had 195 admissions to critical care with a mortality rate of 33.0%.

The insertion of a tracheostomy after around 7–10 days of invasive mechanical ventilation is generally considered a standard of care due to potential to reduce the duration of mechanical ventilation and length of stay on intensive care [6, 7]. The National Confidential Enquiry into Patient Outcome and Death (NCEPOD) study estimated that around 12,000 adult tracheostomies a year were performed in England, Wales and Northern Ireland for such indications in 2014 [8]. Assuming the incidence is the same across Europe (0.2/1,000) then this represents a total of around 100,000 tracheostomies per year. Approximately 70% of these are estimated to be performed by intensivists on intensive care units (ICU) using percutaneous dilatation techniques in the UK, which reflects a shift in practice since the introduction of this technique in 1985 [8].

Genomic analysis of SARS-CoV-2 has determined it to be of a different clade to the betacoronaviruses associated with human severe acute respiratory syndrome (SARS) and Middle East respiratory syndrome (MERS); however, it is well recognised as causing similar respiratory symptoms secondary to a viral pneumonia and in some cases the progression to acute respiratory distress syndrome (ARDS) [4]. The classical radiological appearances are of patchy ground glass opacification and consolidation that may affect multiple lobes and progress to diffuse and dense consolidation. Published studies so far suggest in the early stages of disease there is patchy inflammatory infiltrate, oedema and proteinaceous exudate, with one autopsy study in a patient that died of COVID-19 showing bilateral diffuse alveolar damage with cellular fibromyxoid exudates and interstitial inflammatory infiltrates [9, 10]. The acute lung injury and resultant respiratory failure are responsible for the high levels of invasive mechanical ventilation required for COVID-19 patients.

The COVID-19 pandemic presents unique challenges when considering performing a tracheostomy for such patients for a number of reasons. First, the prognosis of this novel disease is not yet fully understood but mortality rates for those requiring critical care are in the region of 50% [2, 5], which is considerably higher than non-COVID viral pneumonia (22.4%) [5]. This poses a risk of performing futile procedures if a tracheostomy is considered too early. Second, the duration of detectable viral load and correlation with transmission rates during aerosol generating procedures is not yet specifically known. Evidence from China showed infection rates of 3.8% amongst healthcare personnel treating COVID-19 patients [2] but at present, the infection rate related to tracheostomy insertion is unknown. There is no evidence to support a specific technique for tracheostomy insertion in terms of minimising risk of healthcare personnel exposure to airborne droplets.

Whilst post-intubation laryngo-tracheal stenosis is a well-known risk of prolonged endotracheal intubation, there is no evidence it is significantly reduced in patients treated with early tracheostomy (typically less than 10 days). It is too soon to know if there will be any difference amongst the COVID-19 cohort of patients [11, 12]. The incidence of ventilator-associated pneumonia and overall mortality does not improve with early tracheostomy in non-COVID populations [13–17].

Our institution’s experience is that *a significant proportion of *patients admitted for mechanical ventilation are weaning and being successfully extubated between days 5 and 10. This is reflected in our median (IQR) length of stay for survivors of 5 (2.7, 11.8) days and supported by the ICNARC report with a median (IQR) duration of advanced respiratory support of 7 (4, 10) days for 1689 confirmed COVID-19 cases. These are mostly younger patients with less co-morbidity [5]. It is also our experience that those who do not get better at this point may have a poor overall prognosis.

Based upon learning from China and Italy, the basic principles of our local strategies and protocols have surrounded early intubation (Fi0_2_ > 0.4 with increased work of breathing) for younger patients with less co-morbidity and advanced care planning with early decisions regarding ceiling of care for those least likely to derive benefit. Decision-making around co-morbidity and critical care admission is guided by the clinical frailty score in patients older than 65 as recommended by the National Institute for Health and Care Excellence (NICE), and individualised decisions are made for those aged under 65 [18]. This may reduce the overall number of the elderly and those with multiple co-morbidities that would be more likely to require prolonged mechanical ventilation with little or no derived benefit.

Despite this, there will still be a select cohort of patients who will need longer time to wean and where tracheostomy will be indicated. The benefits can include offering a ‘sealed’ system for ongoing respiratory support. This may be preferable to primary extubation with a high risk of failure, a requirement for high-flow oxygen or non-invasive ventilation. Tracheostomy also allows a lower requirement for sedation thereby facilitating less invasive nursing care; fewer infusion pumps, and the potential for their care to be overseen by non-intensive care trained nursing staff [19]. It is well established that shortening the duration of sedation required minimises risk and duration of associated complications, including delirium, and has been shown to reduce the overall length of stay [20].

A tracheostomy in this cohort of patients brings with it a risk to the healthcare professionals involved both during the actual procedure and in the post-procedure period wherein the patients’ tracheostomy needs to be managed.

In a time where healthcare resources are being placed under a huge strain, there is likely to be a proactive but selective role for tracheostomy. This will benefit both the patient, and provide net benefit for population health resources.

The aim of this paper is to review the current international literature to synthesise a proposed practice model, guideline, and describe the pre-emptive setup and training of a dedicated tracheostomy team.

Most importantly, we outline proposed steps that can be taken to minimise aerosol generation for percutaneous tracheostomy in COVID-19 patients, as we could find no guidance in the existing literature describing this.

## Methods

We performed a literature review of tracheostomies during the current and previous pandemics consisting of a PubMed search with the terms tracheostomy and Coronavirus, COVID-19, SARS-CoV-2, SARS, MERS. Papers published between 2000 and April 2020 were critically appraised with respect to specific considerations: timing, viral load, staff safety and technique. In addition all publications referencing Coronavirus, COVID-19 and SARS-CoV-2 were screened for relevant content. All COVID-19-related guidance published from oto-rhino-laryngological societies was also reviewed for relevant content.

## Clinical considerations and risks

### Timing

Evidence from Wuhan demonstrated the median time from hospital admission to death was 5 days [21]. In Lombardy, the median time (IQR) from critical care admission to death has been reported as 7 (5–11) days [22] and in the UK it is currently 6 (3–9) days [5]. Therefore, it seems prudent to wait until the prognosis is clear before doing a futile procedure that could expose healthcare workers to unnecessary risk. The American Academy of Otolaryngology-Head and Neck surgery currently recommends that tracheostomy should not be performed prior to 14 days of endotracheal intubation [23]. Anecdotal reports from colleagues in Spain, Italy and Austria suggest that these countries are also adopting a local policy of around 14 days before undertaking tracheostomy.

Whilst there is no evidence as to the optimal timing of tracheostomy, when considering the current literature with respect to the disease process, the best use of healthcare resources, and staff safety, this time frame appears to be a prudent starting point. Waiting for 14 days will help ensure the indication for the tracheostomy is to treat the ongoing lung injury rather than the effects of the infection itself and is likely to minimise unnecessary interventions.

### Viral load and COVID-19 PCR status

At this stage, there is no evidence to confirm if the viral load of a patient at a specific time point correlates with transmission risk to healthcare workers. It has, however, been shown that viral load does not correlate well with severity of symptoms in an individual, so not all those critically ill will have high viral loads [24].

Initial studies have shown that viral loads from nasal and throat swabs were highest in the early phase of the disease, with clearance by days 9–15 [25, 26]. Evidence from China suggests viral loads in secretions remain detectable up to 2–3 weeks after the onset of symptoms with a median of 20 days and the longest case in their series lasted 37 days [27].

Both the US and Canadian guidelines strongly advise that patients should test negative for COVID-19 before proceeding with tracheostomy [23, 28]. It should be noted that the sensitivity of a single nasal and throat rt-PCR swab for COVID-19 has been estimated at 71% in studies [29], so a negative result should not give false reassurance to surgical teams that exposure risk has passed. In our experience, currently, there are not testing facilities in the UK to allow multiple tests on a single patient, and whilst not definitive, two negative tests may give more reassurance that risk to healthcare personnel is being minimised. In addition, the COVID-19 status may contribute to broader decision-making around proceeding with a tracheostomy. The availability of repeat testing should be a priority for implementation to minimise operator risk.

The evidence thus far in terms of viral load risk would suggest that delaying tracheostomy to at least 14 days post-intubation would represent the safest possible balance. Given the natural disease course, this would likely represent at least 3 weeks since the onset of symptoms. However, it is possible there is an increased risk in the elderly or those with multi-organ failure where viral loads seem to remain elevated for much longer. It has been hypothesised this persistence could be attributed to a lack of appropriate immune response in these patients [27].

### Staff safety

Ensuring minimal exposure and risk to staff performing the procedure will be of paramount importance. Present guidance recommends full personal protective equipment (PPE) for all aerosol-generating procedures including FFP3 mask, eye protection, fluid-repellent disposable surgical gown and gloves [30]. Powered air-purifying respirators (PAPRs) reduce the risk of exposure more than FFP3 masks, but how much more is dependant upon the airflow setting [31]. A literature review of case series and reports from the SARS outbreak identified 23 tracheostomies performed where no healthcare personnel became infected. It was noted that in all cases PAPRs were used by the operating team [32]. Therefore, it appears that this enhanced level of PPE represents the safest possible level of protection for staff and should be mandatory when performing tracheostomy. In our institution, PAPRs are available in ICU and theatres for our tracheostomy team. It is also mandatory for staff to receive PPE training as this can represent a source of contamination if not done correctly [30].

### Procedure setting and location

The ideal location for performing a tracheostomy on a COVID-19 + patient is in a negative pressure side room or operating theatre [33]. This setting would be ideal but not always feasible due to their limited availability and resource implications in a pandemic. Most operating theatres in Europe are positive pressure so do not represent a preferred environment. It may be possible to convert existing theatres to negative pressure rooms after discussion with local estate teams. Moreover, the implications, resources and risks associated with mobilising a ventilated, COVID-19 + patient from the intensive care to the operating theatre should be carefully considered.

Percutaneous tracheostomy (PT) is routinely performed at the bedside in intensive care unit (ICU). Unfortunately, the literature search did not reveal guidance specific to COVID-19 + patients. After discussion with intensivists (DW, ST), it is our policy is to perform PT in the ICU setting in cases where the negative pressure side room will not be available. In the following section, we discuss the precautions of minimising the aerosolization during tracheostomy.

### Technique

In 2016, a systematic review by Brass et al. comparing percutaneous and surgical tracheostomies (ST) did not find any difference in mortality or serious complications, there was, however, a lower rate of wound infections and scarring with PT [34]. There is currently no evidence to advise which procedure is less aerosol generating. However, during the SARS outbreak ST was favoured and felt to be beneficial due to less disruption to ventilation and avoidance of multiple entries to the trachea required with serial dilatations [32]. The single-stage ‘Rhino’ dilator technique is the most common percutaneous technique in the UK, and avoids multiple entries [35].

In addition to the requirement to open the trachea under direct vision, the use of energy devices (e.g. bipolar cautery or ultrasonic shears) to control bleeding during ST can lead to aerosolization [31]. The use of a bronchoscope for guidance in PT has the potential to increase aerosol generation due to the requirement to intermittently open the circuit under positive pressure ventilation. We use a sealed port for bronchoscopy and pause ventilation for insertion and removal to reduce risk of aerosolization. Percutaneous ultrasound is an alternative method of guidance for PT that avoids aerosol generation.

**Fig. 1:**
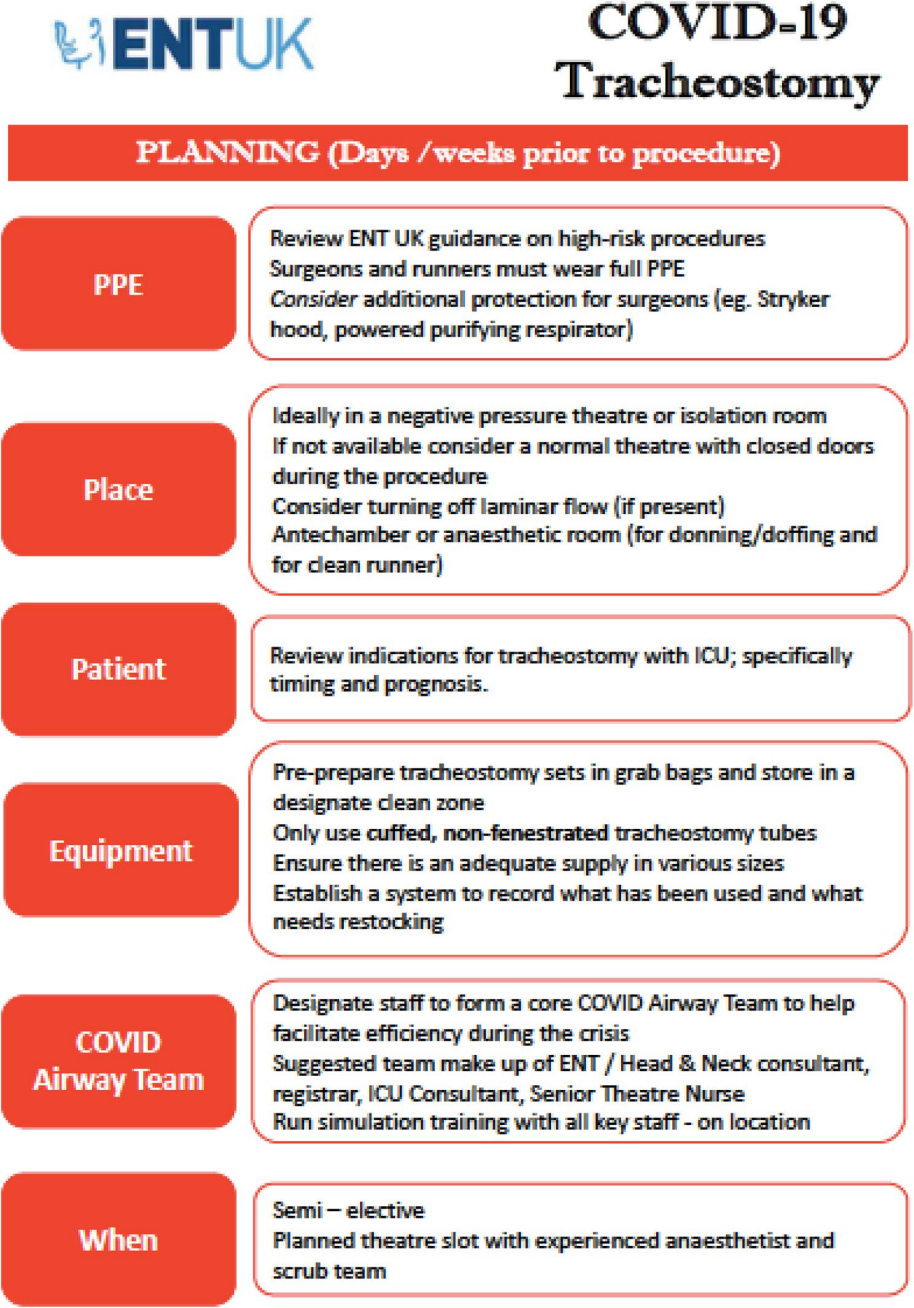

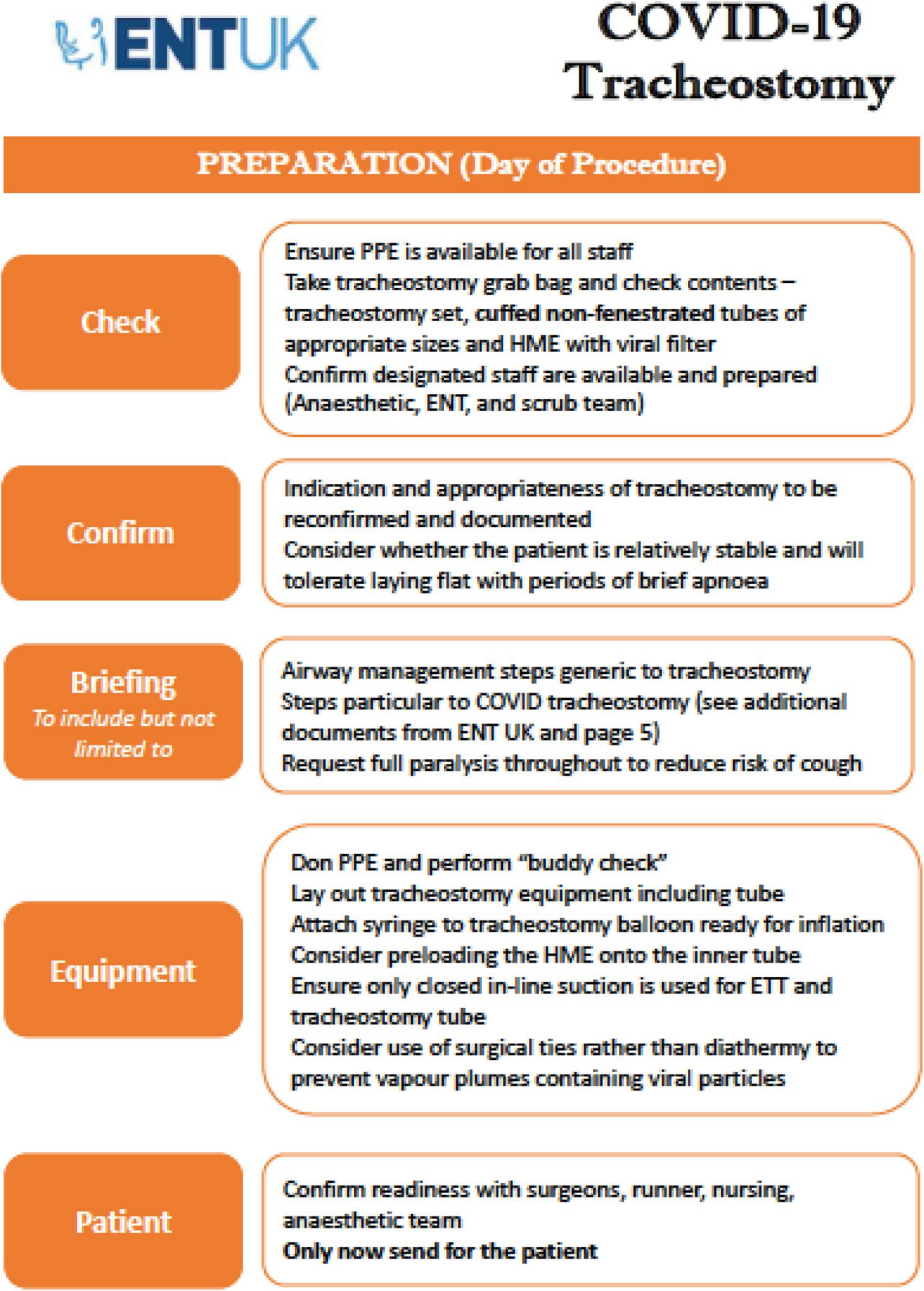

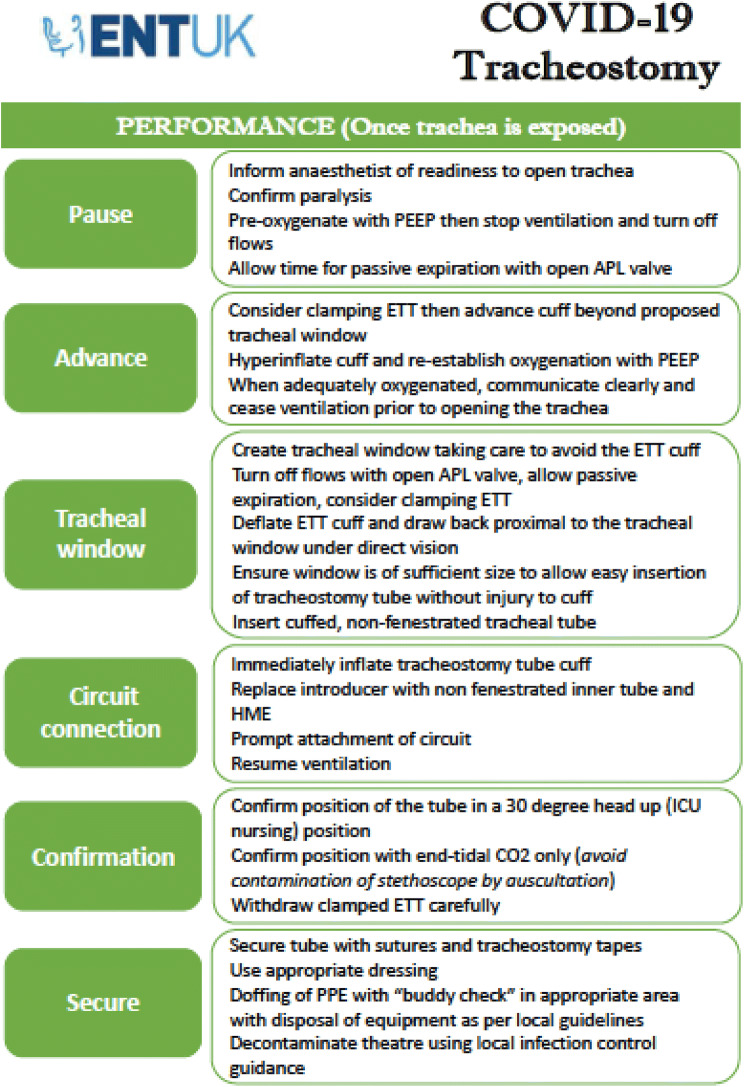

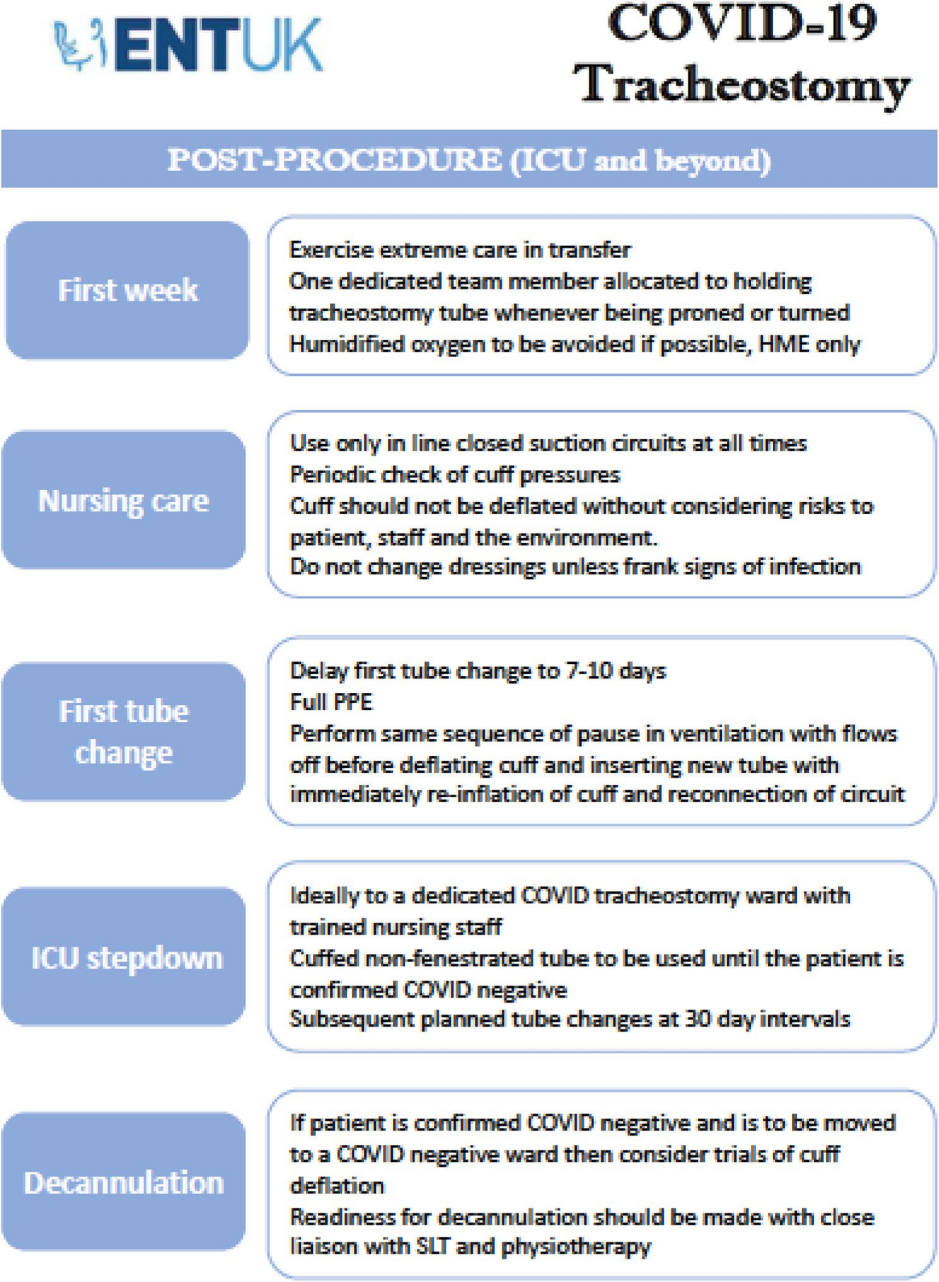
ENT-UK Guidance for COVID-19 tracheostomy. Reproduced with the permission of the authors

Ultimately, the decision on which technique to employ will be guided by local expertise and resources. The ENT-UK guidance set out clear instructions on how to safely perform ST in COVID-19 + patients and it has been cited in multiple other guidelines issued by international bodies since its release [23, 28, 36]. The key actions are summarised in Fig. 1, which is taken from this guidance. The ENT-UK guidance also covers a wide range of considerations valid to both ST and PT concerning planning and post-procedural care.

**Fig. 2:**
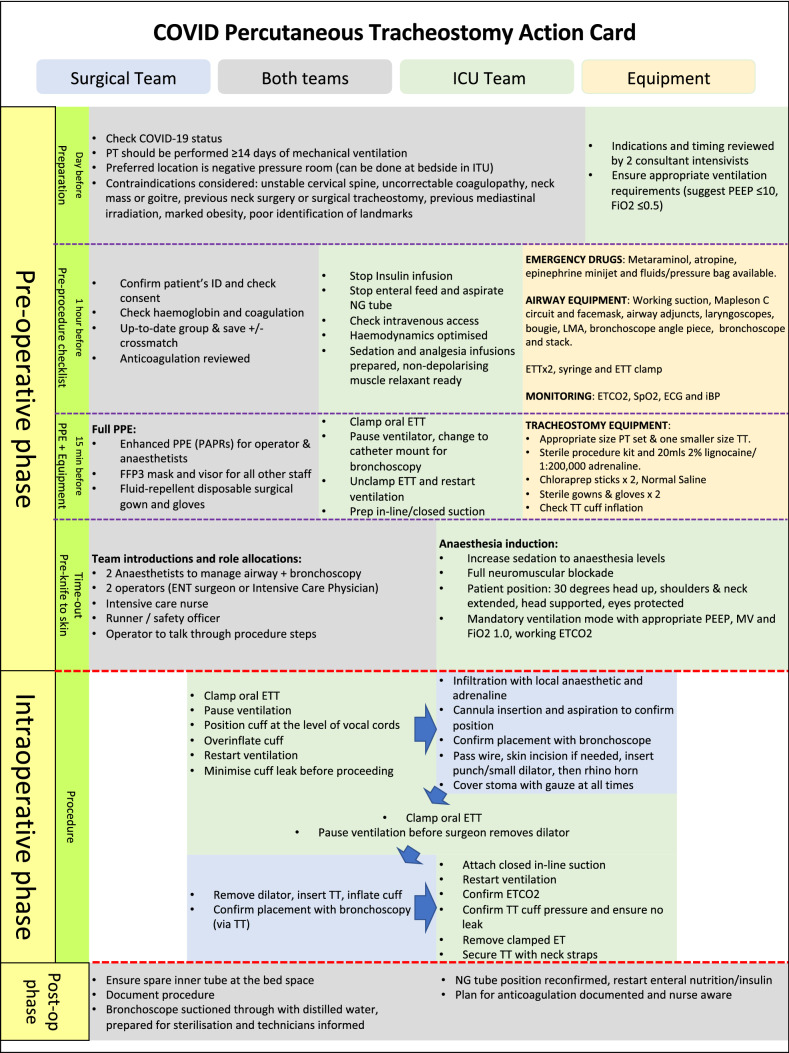
Percutaneous tracheostomy action card

There has been no prior description in the literature of how to minimise aerosol generation in PT. Therefore, we have outlined the considerations in Fig. 2, and some key points are highlighted:

- The use of a bronchoscope is not always necessary, but the use of catheter mount (flexible tube connector) with sealed port for bronchoscopy can minimise aerosol generation if it is required for guidance.
- The procedure is performed under deep sedation and full neuromuscular blockade.
- The initial step is to deflate the endotracheal tube (ETT) cuff and withdraw ETT under laryngoscopic vision until cuff is visualized at the level of the vocal cords. We recommend overinflating cuff to ensure no leak throughout the procedure (as described in ST).
- We advise clamping ETT and pausing ventilation (at end-expiration) during these critical steps that are associated with increased risk of aerosol generation: changing catheter mount, repositioning ETT cuff to the level of the vocal cords, and removal of large rhino dilator.
- Tracheal puncture site should be covered with a swab throughout the procedure to reduce aerosol spread.

## Team training and establishment

ENT-UK has also recommended that dedicated teams be established to carry out these procedures with the specific skills and expertise necessary to perform this as safely and in as timely manner as possible [36]. One consideration for such team is having enough personnel to minimise repeated exposure to high levels of aerosol. A study from the SARS outbreak suggested repeated exposure on consecutive shifts can increase risk of healthcare-associated transmission [37]. We could not find any evidence as to the risk related to the number of procedures performed in a single shift.

With safety at the core of our team-based approach; we have initially set out that our tracheostomy team members will not work more than 1 day per week in this role, and not perform more than two tracheostomies per day. Teams will initially be operational twice a week on non-consecutive days with staff on a rotational basis to minimise exposure whilst ensuring consistency in expertise levels across the week. In addition, all team members will be provided with enhanced PPE to include PAPRs.

In our institution, representation from anaesthetics, intensive care (DW/ST) and otorhinolaryngology-head and neck surgery (ORL-HNS) have formed a cross-speciality working group to design the tracheostomy team and develop the best possible guidance using shared expertise. This will also support multi-disciplinary decision-making around timing and appropriateness of tracheostomy. Within the ORL-HNS department, current levels of expertise and experience for both percutaneous and surgical techniques were established for both consultants and senior trainees to help identify appropriate team leaders and ensure adequate skill levels. Cadaveric refresher training was provided over a 1-day session to 25 personnel from the ORL-HNS department. The team had an opportunity to practice the percutaneous technique in line with our action cards twice each. During this day, we were also able to create an instructional video to support learning and ensure our teams are well rehearsed in the COVID-19 percutaneous technique. A link to the edited video is available here: https://rise.articulate.com/share/7lfMu5dnIBbNMgtS4JIZP96GHFdyEWOH#/.

Our core tracheostomy team consists of

- two anaesthetists to manage airway ± bronchoscopy;
- two operators (ORL-HNS surgeon or intensive care physician);
- intensive care nurse;
- runner/safety officer.

In the event of an emergency, each team will have a nominated leader identified prior to the procedure. Where possible, it is envisaged that PT will be the technique of choice and performed at the bedside. However, in specific circumstances a ST may need to be performed in an operating theatre.

## Indications for tracheostomy

Based on the current literature and our experience the authors have agreed initial selection criteria for use in our institution. These are set out in Table 1.

**Table 1:** Selection criteria for COVID-19 Tracheostomy

Patients may be considered for tracheostomy on or after day 14 of intubation where there is an ongoing requirement for mechanical ventilation
The patient’s case should have been reviewed by at least two intensive care consultants’/senior specialists and the procedure deemed appropriate
Technique and location agreed between intensive care and ORL-HNS team
Ventilation requirements appropriate (suggest Fi0_2_ ≤ 50%, PEEP ≤ 10)
In patients where the prognosis is not clear, they are older (> 70), and/or have multi-organ failure; the decision to proceed should be deferred
Where there are contraindications to intervention (e.g. severe coagulopathy), the decision to proceed to tracheostomy should be delayed further beyond 14 days
Most recent COVID-19 testing status determined (not universally available—priority for implementation)

*Fi0*_*2*_ fraction of inspired oxygen, *PEEP* positive end-expiratory pressure

Guidelines should remain under constant review as higher level evidence of this novel disease and its outcomes from critical care emerge. They propose a robust but concise list to support decision-making in this challenging area.

## Prospective analysis and audit of outcomes

To rapidly learn and improve audit and outcomes of practice will be essential and we propose a mandatory minimum audit dataset in Table 2 that can allow local and regional networks to collaborate to improve the knowledge about what will serve this cohort of patients best.

**Table 2:** Minimum audit dataset

Patient age, sex, co-morbidities, BMI
APACHE II Score
Days post-intubation procedure performed
COVID status at the time of procedure (most recent test result and date)
Technique and location
Members of tracheostomy team and any possible COVID-19 transmission
Procedural complications
Days post-tracheostomy when sedation ceased
Days post-tracheostomy when invasive ventilation ceased (moved to non-invasive ventilation like CPAP)
Days post-tracheostomy discharged from critical care
Days post-tracheostomy discharged from hospital or died
Cause of death (if applicable)
Days to decannulation
Total length of stay

*BMI* body mass index, *APACHE* Acute Physiologic Assessment and Chronic Health Evaluation, *CPAP* continuous positive airways pressure

## Post-procedural care and rehabilitation

The focus of early post-procedural care is to ensure minimisation of aerosol generation risk to healthcare workers and other patients until any risk has passed. Early measures include keeping the cuff inflated, use of in-line suction, and avoidance of humidified oxygen if possible. Cuff deflation, changing of tracheostomy tube and progress on a decannulation protocol should be deferred until the patient is COVID-19 negative where possible. The provision for nursing and rehabilitation of patients once discharged from the ICU will also need to be considered. The cohorting of patients recovering from COVID-19 with tracheostomies onwards with appropriately trained nursing staff is desirable. A number of other considerations and advice for post-procedural care specific to COVID-19 are detailed in the national tracheostomy safety project and ENT-UK guidance [18, 33].

## Summary

These proposals provide a robust framework on which to base delivery of tracheostomy services for critical care units during the COVID-19 pandemic, this can prove an invaluable resource as other countries across Europe and the rest of the world could soon be faced with a surge in demand.

At present, there is no high-level evidence beyond case series upon which to make definitive recommendations, but we have based our proposed guidelines upon consensus from the currently available literature to form a pragmatic and safe approach. There is no doubt that as more rapidly emerging higher level evidence becomes available, our recommendations will be refined and improved.

The decision to perform tracheostomy in these patients requires careful consideration, planning and regular scrutiny if it is going to be of net benefit to patients and critical care services in terms of optimising healthcare resource utilisation, ensuring patient and staff safety and providing optimal long-term outcomes.

Urgent planning, training and collaborative data collection will be vital, and ORL-HNS surgeons have the potential to offer a valuable role in supporting critical care teams with this service at a very challenging time.

## Key recommendations

- Performing tracheostomy for prolonged invasive mechanical ventilation on COVID-19 must be very carefully considered.
- The mortality for patients ventilated with COVID-19 is around 50% and tracheostomy should not be performed until the prognosis is deemed favourable.
- The current literature and consensus opinion suggest that tracheostomy should only be considered after 14 days of invasive mechanical ventilation when the patient is still not suitable for extubation.
- All staff performing tracheostomy in COVID-19 patients must be equipped with enhanced PPE including PAPRs.
- There is currently no evidence whether percutaneous or surgical tracheostomy is less aerosol generating.
- Deep sedation, complete neuromuscular blockade and endotracheal tube cuff hyperinflation should be employed for all tracheostomy procedures.
- Strategies to reduce aerosol generation in percutaneous tracheostomies include avoiding the use of bronchoscope (or using catheter mount with sealed port), pausing ventilation at end-expiration and clamping the endotracheal tube before key steps in the procedure (changing catheter mount, withdrawing tube, removing dilators, inserting tracheostomy tube).
- Early planning and staff training is essential and can ensure adequate resources and expertise to support the increase in demand whilst minimising exposure risk for healthcare personnel.
- Prospective analysis and audit of outcomes are essential to rapidly learn and improve outcomes for this cohort of patients.
